# Nutritional Aspects in Inflammatory Bowel Diseases

**DOI:** 10.3390/nu12020372

**Published:** 2020-01-31

**Authors:** Paola Balestrieri, Mentore Ribolsi, Michele Pier Luca Guarino, Sara Emerenziani, Annamaria Altomare, Michele Cicala

**Affiliations:** Unit of Gastroenterology, Campus Bio-Medico University, 00128 Rome, Italy; m.ribolsi@unicampus.it (M.R.); m.guarino@unicampus.it (M.P.L.G.); s.emerenziani@unicampus.it (S.E.); a.altomare@unicampus.it (A.A.); m.cicala@unicampus.it (M.C.)

**Keywords:** IBD, CD, UC, malnutrition, enteral nutrition, malabsorption

## Abstract

Crohn’s disease (CD) and ulcerative colitis (UC) are chronic, relapsing, inflammatory disorders of the digestive tract that characteristically develop in adolescence and early adulthood. The reported prevalence of malnutrition in inflammatory bowel disease (IBD) patients ranges between 20% and 85%. Several factors, including reduced oral food intake, malabsorption, chronic blood and proteins loss, and intestinal bacterial overgrowth, contribute to malnutrition in IBD patients. Poor nutritional status, as well as selective malnutrition or sarcopenia, is associated with poor clinical outcomes, response to therapy and, therefore, quality of life. The nutritional assessment should include a dietetic evaluation with the assessment of daily caloric intake and energy expenditure, radiological assessment, and measurement of functional capacity.

## 1. Introduction:

Crohn’s disease (CD) and ulcerative colitis (UC) are chronic, relapsing, inflammatory disorders of the digestive tract that characteristically develop in adolescence and early adulthood. The reported prevalence of malnutrition in inflammatory bowel disease (IBD) patients ranges between 20% and 85% [1,2]. Several studies have reported a prevalence of weight loss in 70%–80% of hospitalized IBD patients and in 20%–40% of outpatients with CD [3,4].

Malnutrition can occur both in UC and in CD, but the prevalence of protein–energy and specific nutrient malnutrition seems to be higher in CD compared to UC, probably because it can affect any part of the gastrointestinal tract and, mainly, the small bowel [1].

The mechanisms underlying malnutrition in inflammatory bowel disease include reduced oral food intake, malabsorption of nutrients, enteric nutrient loss, increased energy requirements due to systemic inflammation and, occasionally, iatrogenic factors (drug- and surgery-related). The assessment of the nutritional status and the need of supportive nutritional therapy play a pivotal role in the clinical care of inflammatory bowel disease patients.

In this review article, we aim to focus on the role of nutrition in inflammatory bowel disease patients.

## 2. Mechanisms of Malnutrition in IBD

Several factors contribute to malnutrition in IBD patients. It is known that a reduced oral food intake is a main determinant of malnutrition in patients with IBD. Several mechanisms are involved in the reduction of food intake. Patients with active IBD often experience loss of appetite due to nausea, vomiting, abdominal pain, and diarrhea. Medications may also induce nausea, vomiting, or anorexia [5,6]. Glucocorticoids often reduce phosphorus, zinc, and calcium absorption and may lead to osteoporosis. Long-term sulfasalazine therapy, a folic acid antagonist, might be related to anemia. Hospitalization itself or prolonged restrictive diet may lead to a significant reduction of food intake [7,8].

Malabsorption is, among other factors, strongly related to mucosal alterations, such as impaired epithelial transport and loss of epithelial integrity. It has been reported that ileal involvement in CD patients plays a relevant role in reducing nutrients absorption [9]. In particular, alterations of ionic transport cause the loss of electrolytes and fluids. Moreover, active inflammation leads to a chronic loss of blood and proteins within the intestinal lumen.

Small intestinal bacterial overgrowth is a hallmark of IBD patients and is related, among several factors, to chronic intestinal inflammation or surgical removal of the ileocecal valve. Small intestinal bacterial overgrowth may contribute to increased intestinal permeability [10,11,12,13,14,15]. It has been demonstrated that bacterial overgrowth and increased intestinal motility play a key role in energy intake [16,17]. In particular, a small intestinal bacterial overgrowth reduces the digestion and absorption of nutrients in addition to producing osmotically active metabolites that contribute to discomfort and diarrhea. The consequent accelerated gastrointestinal transit reduces the contact time of the luminal contents with the mucosal surface and, therefore, leads to malabsorption.

Surgery plays a pivotal role in determining nutrients malabsorption by inducing diarrhea across several mechanisms. The resection of large small-bowel segments is usually followed by reduced digestion and absorption of nutrients and, therefore, by watery diarrhea. Moreover, a large ileal resection leads to reduced intestinal uptake of bile acids and, therefore, causes bile salt diarrhea [18,19,20,21]. It has been proved that a surgical resection of the ileum of more than 100 cm leads to a loss of bile acids which exceeds the rate of hepatic synthesis [22]. As a consequence of the depletion of the bile acid pool, fat digestion and absorption is impaired, thus determining steatorrhea [23].

**Figure 1:**
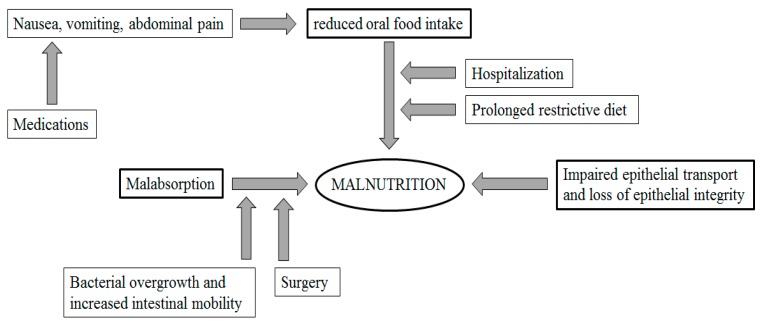
Mechanisms of malnutrition in inflammatory bowel disease (IBD) patients.

Chronic bowel inflammation or intestinal surgery may accelerate the intestinal transit. A rapid gastrointestinal transit may limit the contact time of the luminal contents with the mucosal surface, thus leading to malabsorption and resulting in greater stool volume and diarrhea. Mechanisms of malnutrition in IBD patients are depicted in Figure 1.

## 3. Clinical Aspects of Malnutrition in Patients with IBD

Malnutrition is a common condition among IBD patients and can be recognized by protein–energy malnutrition, an altered body composition, and micronutrient deficiencies. During the course of the disease, protein–energy malnutrition and deficiencies of specific nutrients may represent clinical concerns in IBD patients, and their prevention is essential to avoid complications.

Malnutrition is one of the most important factors associated with a poor clinical outcome in patients with IBD [24,25]. The severity of malnutrition in IBD patients is dependent on the activity, duration, and extent of the disease and, in particular, on the magnitude of the inflammatory systemic response mediated by pro-inflammatory cytokines such as tumor necrosis factor (TNF)-α and interleukins-1 and -6, which can increase catabolism and lead to anorexia [26].

Among IBD patients, significant differences in the nutritional status between CD and UC patients have been described, since the involvement of the small intestine is accompanied by a higher incidence of protein–energy malnutrition and micronutrient and/or vitamin deficiencies [4]. Moreover, patients with CD generally develop malnutrition over a long period of time, whereas patients with UC tend to present a precipitous nutritional deficiency during a severe acute flare of the disease or in case of hospitalization. [27]

Another significant aspect of malnutrition in IBD is correlated with alterations of the body composition, in particular of the ratio between fat mass (FM), consisting of both visceral and subcutaneous adipose tissues, and lean mass, also called fat-free mass (FFM). The altered body composition in IBD patients may impact on the course of the disease, on the responsiveness to IBD treatments, on the outcomes of surgery, and on the quality of life. [28] In the last decades, the depletion of muscle mass has emerged as a crucial variable in the nutritional assessment in many chronic inflammatory conditions, such as cirrhosis [29,30], cryptogenic organizing pneumonia [31], and IBD. A progressive and generalized loss of lean muscle mass associated with decreased muscle strength or physical performance characterizes a syndrome, defined sarcopenia, that significantly impact on quality of life and causes physical disability [32]

A systematic review reported that up to 60% IBD patients have decreased muscle mass when compared with healthy subjects [33]. Interestingly, Adams et al. showed that a significant proportion of IBD patients affected by sarcopenia (41.5%) presented a normal body mass index (BMI) and, therefore, would not be identified as undernourished by traditional measures. Moreover, 20% of sarcopenic patients in this cohort were overweight or obese. [34] Sarcopenia has been associated with an increased need for surgery and poor surgical outcomes in IBD, as well as with osteopenia [34,35,36]. This highlights the need for malnutrition and/or sarcopenia screening in all IBD patients, not just those who appear undernourished.

The most common micronutrient deficiencies in IBD patients concern iron, calcium, selenium, zinc, magnesium, water-soluble vitamins, in particular, B12 and folic acid, and fat-soluble vitamins, such as A, D, and K [37,38].

Anemia is the most frequent systemic complication and extra-intestinal manifestation of IBD [39,40,41]. In the management of IBD patients, the distinction between anemia due to iron deficiency and anemia due to chronic disease is essential, even if both conditions typically overlap. Iron deficiency is the main cause of anemia in the adult IBD population, with a prevalence between 36% and 90% [42]. The main factors determining iron deficiency in IBD are: inadequate iron intake in the diet, active inflammation leading to a continuous blood loss from the ulcerated surface of the bowel, impaired iron utilization due to the systemic inflammatory status, and impaired iron uptake through the duodeno–jejunal mucosa [43]. According to European Crohn’s and Colitis Organisation (ECCO) guidelines, the diagnostic criteria for iron deficiency depend on the severity of inflammation. In patients without any evidence of active disease, a serum ferritin level <30 µg/L is an appropriate criterion to diagnose anemia, while a serum ferritin level up to 100 µg/L may still be consistent with iron deficiency in the presence of active inflammation, since the serum ferritin levels can be high despite the iron stores being empty. Unlike ferritin, chronic inflammation has no effect on soluble transferrin receptor (sTfR) levels, and the concentration of sTfR in the serum may be considered an indicator of the supply of iron available for erythropoiesis [44].

Low bone mass and osteoporosis are common in both male and female patients with IBD [20%–50%]. Contributing factors include overall cumulative corticosteroid exposure, extensive small-bowel disease or resection, chronic inflammation, lack of physical activity, and deficiencies of calcium, vitamins, and other micronutrients [36]. The prevalence of calcium deficiency among IBD patients is approximately 13% in CD patients and 10% in UC patients, whereas up to 70% of CD patients and up to 40% of UC patients present low levels of vitamin D. [45] The reduction of the intestinal absorptive surface after a resection of the jejunum or ileum tract resulting in vitamin D deficiency, the binding of calcium to unabsorbed fatty acids in the intestinal lumen, or a restrictive diet excluding milk and dairy products may be involved in the pathogenesis of calcium deficiencies in IBD patients. Therefore, in active IBD patients and, particularly, in patients undergoing treatment with steroids, serum calcium and 25(OH) vitamin D should be monitored and supplemented, if required, to help prevent low bone mineral density and, moreover, because of the potential therapeutic role of 25(OH) vitamin D. Several studies have proved, indeed, the immune modulating effects of vitamin D in the IBD population. Vitamin D appears to have an important role in innate immunity as well as in adaptive immunity. Therefore, it has been suggested that vitamin D deficiency may be involved in the pathogenesis of IBD [46,47]. In a 22-year follow-up prospective study, higher predicted plasma 25(OH)D levels were associated with a significant reduction in the risk of incident CD but not of UC [48].

The water-soluble B vitamin folate is an essential vitamin for humans and is obtained from the diet, especially from fruits and vegetables. Folic acid deficiency is a common condition in IBD patients, with a prevalence varying from 8.8% in UC patients to 28.8% in CD patients [49]. Potential determinants of folate deficiency include inadequate intake and malabsorption due to the loss of intestinal surface area as a consequence of active inflammation, resection, or fistulas. Folate deficiency may be also caused by therapeutic agents, such as sulfasalazine and methotrexate, that can inhibit folate absorption. Therefore, acid folic deficiency can cause anemia, and its dosage has to be included in the work-up assessment of anemia in the IBD population [1]. Moreover, folic acid is an essential co-factor in the metabolism of homocysteine–methionine, and folate deficiency may lead to hyperhomocysteinemia, which is an established risk factor for many cardiovascular disease and may be responsible of the increased incidence of arterial and venous thromboembolic events observed in CD and UC patients [50]. Finally, folate deficiency is also known as an established risk factor of colorectal cancer in the IBD population [51].

It is well established that vitamin B12 deficiency is more common in patients with CD than in those with UC, since the absorption of vitamin B12 requires an intact ileum to absorb the intrinsic factor–cobalamin complex [52]. Previous studies have reported that CD patients who underwent resection of more than 60 cm of terminal ileal will develop B12 deficiency, highlighting the need to carefully monitor these patients, who will require a lifelong B12 replacement [53]. Therefore, screening for vitamin B12 deficiency in the IBD population, especially in patients with post-operative CD, is necessary in order to avoid related clinical complications, such as megaloblastic anemia and peripheral neuropathy, and to prevent hyperhomocysteinemia, an independent risk factor for thromboembolism [54]. The British Society of Gastroenterology guidelines suggest B12 replacement for all patients with ileal resection greater than 20 cm and yearly monitoring of B12 levels for patients with ileal resection below 20 cm [55]. The frequency of nutritional deficiencies in CD and UC patients is reported in Table 1.

## 4. Overview of Nutritional Assessment

The nutritional assessment should include a dietetic evaluation with the assessment of daily caloric intake and energy expenditure, radiological assessment, and measurement of functional capacity.

According to the World Health Organization standardized criteria, patients are considered well nourished when their BMI is between 18.5 and 24.5 kg/m^2^, underweight or malnourished when their BMI is ≤18.5 kg/m^2^, and overweight when their BMI is ≥25 kg/m^2^ [57].

Several methods can be employed to reach a full characterization of the body composition, although there is no standard radiological measure of lean muscle mass [58].

Computerized tomography and magnetic resonance provide a radiological measurement of muscle mass: skeletal muscle index (SMI) at the level of the third lumbar vertebral body, cross-sectional area of the psoas muscles (the total psoas area index), or psoas muscle density using mean Hounsfield unit average calculations (HUAC) are indirect indicators of the sarcopenic status [33]. However, they have a limited use in clinical practice due to their high cost.

Nowadays, dual-energy X-ray absorptiometry (DEXA) and bioelectrical impedance analysis (BIA) are considered rapid and non-invasive techniques to define sarcopenia. DEXA provides an assessment of FM, FFM, and bone mineral density and is considered the gold standard to assess body composition. It employs an energy source that produces photons at two different energy levels, 40 and 70 keV, which pass through tissues, attenuating at rates related to tissues’ elemental composition. Bone is rich in highly attenuating phosphorous, calcium, and minerals and is easily distinguished from soft tissues, such as adipose tissue and muscle [59].

BIA is a simple, non-invasive, and reproducible technique that indirectly estimates body composition using prediction equations, through the measurement of body reactance and resistance combined with anthropometric variables (weight and height), sex, and age [60]. Resistance depends on the levels of electrolyte and water in a tissue: lean tissues are good conductors, since they contain water and electrolytes, while fatty tissues and bones are poor conductors. Reactance is the measure of the opposition that a circuit, i.e., all biological tissues, presents to an electric current, which, in the body, is due to the condenser-like properties of the cells. Lean body mass has high reactance (high water content), while fat mass has low reactance (low water content).

The quality of lean tissue, particularly skeletal muscle, is evaluated through functional measurements. It has been reported that the strength of a muscle deteriorates more quickly than its mass, suggesting that muscle strength may be an early indicative parameter of muscle health [61,62,63]. Muscle function can be clinically investigated to aid in the diagnosis of sarcopenia by measuring either hand grip strength, using a standard dynamometer, or gait speed [58]. These measures, in addition to body composition assessments, could be useful to better understand the metabolic and functional integrity of the muscle mass.

## 5. Role of Diet in IBD

Although several studies demonstrate improved disease activity and prolonged time to relapse following precise dietary regimens, the efficacy of many of these diet protocols has not been fully clarified. A mainstay of the dietary protocols in patients with IBD is the low-residue diet (<10–15 g/day of fiber), especially for those patients at risk of gastrointestinal obstruction [64,65]. However, evidence for the efficacy of low-residue diets in IBD is lacking. An Italian study evaluating 70 patients with non-stenosing CD, randomly assigned to follow a low-residue diet (~3 g/day of fiber) or a normal Italian diet (13 g/day of fiber) for a mean of 29 months, reported that there was no difference between the two groups in clinical outcome, including symptoms, need for hospitalization, need for surgery, new complications, nutritional status, or postoperative disease recurrence [66].

It has been hypothesized that many types of fiber may have an important effect on the gut microbiota and thus possibly on the maintenance of remission in IBD patients. A prospective longitudinal cohort study of patients with CD (*n* = 1130) and UC (*n* = 489) in remission examined the association between fiber exposure and the risk of disease flare [67]. CD patients with higher fiber intake were 40% less likely to experience a flare at six months than those with lower fiber consumption. On the other hand, fiber intake in patients with UC did not have any impact [68]. Nowadays, several exclusion diet plans are gaining interest for IBD treatment. A low-FODMAP (Fermentable Oligosaccharides, Disaccharides, Monosaccharides and Polyols) diet has been proposed for reducing the impact of symptoms in patients with controlled IBD without active inflammation. It has been reported that la ow-FODMAP diet is able to reduce the impact of symptoms (e.g., abdominal pain, belching, bloating, flatulence, incomplete evacuation, nausea, and heartburn) in 78% of IBD patients [69].

The specific carbohydrate diet is a novel diet approach based on the restriction of complex carbohydrates and refined sugar. The rationale of this diet plan is that sugars and complex carbohydrates are malabsorbed and could cause alterations in the microbiome composition, contributing to the intestinal inflammation of IBD [70]. However, the effect of the specific carbohydrate diet on the clinical course of IBD remains to be validated.

Therefore, up to now, no oral diet in IBD can be generally recommended to promote remission in IBD patients with active disease.

## 6. Nutritional Treatment in the Management of IBD

Enteral nutrition (EN) therapy, with oral nutritional supplements or tube feeding, is aimed at maintaining or restoring the nutritional status of individuals characterized by reduced oral intake. In routine practice, EN should be administered in some circumstances, i.e., severe malnutrition, moderate malnutrition with food intake expected to be insufficient for >5 days, ordinary nutritional status with insufficient food intake >10 days, or moderate/severe hypercatabolism [71,72]. It is widely accepted that EN is preferable over parenteral nutrition (PN), because it is associated with a lower incidence of complications and lower costs. Moreover, luminal nutrients are currently considered as basic trophic factor for the intestinal mucosa, also preventing bacterial translocation and preserving the gastrointestinal function.

Several studies have demonstrated the efficacy of EN in active CD patients, although its mechanisms of action remain unknown. Is has been speculated that nutrients are able to modulate the commensal microflora and the intestinal immune response by reducing antigen exposure. Consequently, EN seems to exert an anti-inflammatory effect on the intestinal mucosa by reducing IL-6 production and increasing the production of insulin-like growth factor (IGF)-1 [73]. It has been shown that EN is effective in the treatment of the acute phase of CD, with remission rates ranging from 20% to 84.2%, regardless of disease location [74]. However, it still remains to be fully elucidated whether EN is actually an effective therapeutic approach to CD. In this respect, results emerging from the literature are controversial. It has been demonstrated that corticosteroids are more effective than EN for treating active CD [75,76]. On the other hand, other studies report that steroids are as effective as EN [77,78].

Exclusive EN might be considered a first-line therapy in children, because of its good efficacy in prompting remission, its beneficial effects on growth, and its limited adverse effects. However, further studies on different enteral formulations (elemental, immuno-nutrition, gut-specific nutrients) and on the combination of enteral and drug therapy are needed [4].

Enteral feeding should always take preference over parenteral therapy, unless it is completely contraindicated, such as in intestinal ischaemia, ileus, severe shock condition, high-output intestinal fistulas, or severe intestinal haemorrhage [79]. One of the most relevant indications for PN in IBD patients is short bowel syndrome with severe malabsorption of nutrients and/or fluid that cannot be managed with enteral feeding. PN is also indicated in patients with obstructive disease, when there is no possibility of placement of a feeding tube beyond the obstruction segment or when this procedure has failed. In the perioperative period, nutritional support should be early administrated because, independently of the route of administration, it decreases the risk of postoperative complications [80].

The successful use of PN requires proper selection of patients and awareness of its complications, such as metabolic alterations requiring a daily nutritional monitoring or related to the catheter itself.

## 7. Conclusions

Nutritional aspects in IBD are particularly relevant as they potentially influence disease activity and, therefore, its morbidity. Results from the currently available literature show that a poor nutritional status, as well as selective malnutrition or sarcopenia, is associated with poor clinical outcomes, response to therapy, and, therefore, quality of life. Several factors contribute to malnutrition in IBD patient. The nutritional assessment should include a dietetic evaluation with the assessment of daily caloric intake and energy expenditure, radiological assessment, and measurement of the functional capacity. EN might be considered a first-line therapy in children, because it has good efficacy in prompting remission, beneficial effects on growth, and few adverse effects.

In conclusion, a multidisciplinary assessment of patients with IBD is always encouraged, and nutrition strategies should always be tailored for each patient.

## Figures and Tables

**Table 1:** Frequency of nutritional deficiencies in Crohn’s disease (CD) and ulcerative colitis( UC) patients (adapted from Lochs) [56].

	Frequency	
	CD	UC
Weight loss	65%–75%	18%–62%
Anemia	60%-80%	66%
Iron deficiency	39%	81%
Vitamin B_12_ deficiency	48%	5%
Folic acid deficiency	54%	36%
Calcium deficiency	13%	ND
Vitamin D deficiency	75%	ND
Magnesium deficiency	14%–33%	ND
Vitamin K deficiency	ND	ND

ND: not defined.

## References

[1] Goh J., O’Morain C.A. (2003). Review article: Nutrition and adult inflammatory bowel disease. Aliment. Pharmacol. Ther..

[2] Clare F., Donnellan L.H., Simon L. (2013). Nutritional management of Crohn’s disease. Ther. Adv. Gastroenterol..

[3] Lanfranchi G.A., Brignola C., Campieri M., Bazzocchi G., Pasquali R., Bassein L., Labo G. (1984). Assessment of nutritional status in Crohn’s disease in remission or low activity. Hepatogastroenterology.

[4] Hartman C., Eliakim R., Shamir R. (2009). Nutritional status and nutritional therapy in inflammatory bowel diseases. World J. Gastroenterol..

[5] Hanauer S.B., Stathopoulos G. (1991). Risk benefit assessment of drugs used in the treatment of inflammatory bowel disease. Drug Saf..

[6] Singleton J.W., Law D.H., Kelley M.L., Mekhjian H.S., Sturdevant R.A. (1979). National Cooperative Crohn’s Disease Study: Adverse reactions to study drugs. Gastroenterology.

[7] H’ebuterne F.J., Al-Jaouni R., Schneider S. (2009). Nutritional consequences and nutrition therapy in Crohn’s disease. Gastroenterol. Clin. Biol..

[8] Lucendo A.J., De Rezende L.C. (2009). Importance of nutrition in inflammatory bowel disease. World J. Gastroenterol..

[9] Capristo E., Addolorato G., Mingrone G., Greco A.V., Gasbarrini G. (1998). Effect of disease localization on the anthropometric and metabolic features of Crohn’s disease. Am. J. Gastroenterol..

[10] Morris T.H., Sorensen S.H., Turkington J., Batt R.M. (1994). Diarrhoea and increased intestinal permeability in laboratory beagles associated with proximal small intestinal bacterial overgrowth. Lab. Anim..

[11] MacMahon M., Lynch M., Mullins E. (1994). Small intestinal bacterial overgrowth—An incidental finding?. J. Am. Geriatr. Soc..

[12] May G.R., Sutherland L.R., Meddings J.B. (1993). Is small intestinal permeability really increased in relatives of patients with Crohn’s disease?. Gastroenterology.

[13] Hollander D. (1993). Permeability in Crohn’s disease: Altered barrier functions in healthy relatives?. Gastroenterology.

[14] Riordan S.M., Mclver C.J., Thomas D.H., Duncombe V.M., Bolin T.D., Thomas M.C. (1997). Luminal bacteria and small-intestinal permeability. Scand J. Gastroenterol..

[15] Saltzman J.R., Russell R.M. (1994). Nutral consequences of intestinal bacterial overgrowth. Compr.Ther..

[16] Hoverstad T., Bjorneklett A., Fausa O., Midtvedt T. (1985). Shortchain fatty acids in the small-bowel bacterial overgrowth syndrome. Scand J. Gastroenterol..

[17] Fromm H., Malavolti M. (1986). Bile acid-induced diarrhoea. Clin. Gastroenterol..

[18] Hafkenscheid J.C. (1977). Influence of bile acids on the (Na+-K+)-activated- and Mg2+-activated ATPase of rat colon. Pflugers. Arch. Eur. J. Physiol..

[19] Kirwan W.O., Smith A.N., Mitchell W.D., Falconer J.D., Eastwood M.A. (1975). Bile acids and colonic motility in the rabbit and the human. Gut.

[20] Krag B., Krag E. (1976). Regional ileitis (Crohn’s disease). II. Electrolyte and water movement in the ileum during perfusion with bile acids. Scand J. Gastroenterol..

[21] Hofmann A.F., Poley J.R. (1972). Role of bile acid malabsorption in pathogenesis of diarrhea and steatorrhea in patients with ileal resection. I. Response to cholestyramine or replacement of dietary long chain triglyceride by medium chain triglyceride. Gastroenterology.

[22] Hofmann A.F., Poley J.R. (1969). Cholestyramine treatment of diarrhea associated with ileal resection. N. Engl. J. Med..

[23] Borgstrom B., Lundh G., Hofmann A. (1963). The site of absorption of conjugated bile salts in man. Gastroenterology.

[24] Jansen I., Prager M., Valentini L., Büning C. (2016). Inflammation-driven malnutrition: A new screening tool predicts outcome in Crohn’s disease. Br. J. Nutr..

[25] Takaoka A., Sasaki M., Nakanishi N., Kurihara M., Ohi A., Bamba S., Andoh A. (2017). Nutritional Screening and Clinical Outcome in Hospitalized Patients with Crohn’s Disease. Ann. Nutr. Metab..

[26] Cabré E., Gassull M.A. (2001). Nutrition in inflammatory bowel disease: Impact on disease and therapy. Curr. Opin. Gastroenterol..

[27] Han P.D., Burke A., Baldassano R.N., Rombeau J.L., Lichtenstein G.R. (1999). Nutrition and inflammatory bowel disease. Gastroenterol. Clin. N. Am..

[28] Bryant R.V., Trott M.J., Bartholomeusz F.D., Andrews J.M. (2013). Systematic review: Body composition in adults with inflammatory bowel disease. Aliment. Pharmacol. Ther..

[29] Tandon P., Raman M., Mourtzakis M., Merli M. (2017). A Practical Approach to Nutritional Screening and Assessment in Cirrhosis. Hepatology.

[30] Seong H.K., Woo K.J., Soon K.B., Seung H.C., Moon Y.K. (2018). Impact of sarcopenia on prognostic value of cirrhosis: Going beyond the hepatic venous pressure gradient and MELD score. J. Cachexia Sarcopenia Muscle.

[31] Anna E.B., Nilay H., Samantha K., Matthew M. (2017). Sarcopenia and frailty in chronic respiratory disease. Chron Respir. Dis..

[32] Biolo G., Cederholm T., Muscaritoli M. (2014). Muscle contractile and metabolic dysfunction is a common feature of sarcopenia of aging and chronic diseases: From sarcopenic obesity to cachexia. Clin. Nutr..

[33] Ryan E., McNicholas D., Creavin B., Kelly M.E., Walsh T., Beddy D. (2019). Sarcopenia and Inflammatory Bowel Disease: A Systematic Review. Inflamm. Bowel Dis..

[34] Adams D.W., Gurwara S., Silver H.J. (2017). Sarcopenia is common in overweight patients with inflammatory bowel disease and may predict need for surgery. Inflamm. Bowel Dis..

[35] Pedersen M., Cromwell J., Nau P. (2017). Sarcopenia is a predictor of surgical morbidity in inflammatory bowel disease. Inflamm. Bowel Dis..

[36] Bryant R.V., Ooi S., Schultzetal C.G. (2015). Low muscle mass and sarcopenia: Common and predictive of osteopenia in inflammatory bowel disease. Aliment. Pharmacol. Ther..

[37] Weisshof R., Chermes I. (2015). Micronutrient deficiencies in inflammatory bowel disease. Curr. Opin. Clin. Nutr. Metab Care.

[38] Hwang C., Ross V., Mahadevan U. (2012). Micronutrient deficiencies in inflammatory bowel disease: From A to zinc. Inflamm. Bowel Dis..

[39] Gasche C. (2000). Anemia in IBD: The overlooked villain. Inflamm. Bowel Dis..

[40] Kulnigg S., Gasche C. (2006). Systematic review: Managing anemia in Crohn’s disease. Aliment. Pharmacol. Ther..

[41] Gisbert J.P., Gomollon F. (2008). Common misconceptions in the diagnosis and management of anemia in inflammatory bowel disease. Am. J. Gastroenterol..

[42] Goodhand J.R., Kamperidis N., Rao A., Laskaratos F., McDermott A., Wahed M., Naik S., Croft N.M., Lindsay J.O., Sanderson I.R. (2012). Prevalence and management of anemia in children, adolescents, and adults with inflammatory bowel disease. Inflamm. Bowel Dis..

[43] Gomollón F., Gisbert J.P. (2009). Anemia and inflammatory bowel diseases. World J. Gastroenterol..

[44] Dignass A.U., Gasche C., Bettenworth D., Birgegård G., Danese S., Gisbert J.P., Gomollon F., Iqbal T., Katsanos K., Koutroubakis I. (2015). European consensus on the diagnosis and management of iron deficiency and anaemia in inflammatory bowel diseases. J. Crohns Colitis.

[45] Massironi S., Rossi R.E., Cavalcoli F.A., Della Valle S., Fraquelli M., Conte D. (2013). Nutritional deficiencies in inflammatory bowel disease: Therapeutic Approaches. Clin. Nutr..

[46] Cantorna M.T., Zhu Y., Froicu M., Wittke A. (2004). Vitamin D status, 1,25-dihydroxyvitamin D3, and the immune system. Am. J. Clin. Nutr..

[47] Cantorna M.T., Mahon B.D. (2005). D-hormone and the immune system. J. Rheumatol. Suppl..

[48] Ananthakrishnan A.N., Khalili H., Higuchi L.M., Bao Y., Korzenik J.R., Giovannucci E.L., Richter J.M., Fuchs C.S., Chan A.T. (2012). Higher predicted vitamin d status is associated with reduced risk of Crohn’s disease. Gastroenterology.

[49] Yakut M., Ustün Y., Kabaçam G., Soykan I. (2010). Serum vitamin B12 and folate status in patients with inflammatory bowel diseases. Eur. J. Intern. Med..

[50] Talbot R.W., Heppell J., Dozois R.R., Beart R.W. (1986). Vascular complications of inflammatory bowel disease. Mayo Clin. Proc..

[51] Pohl C., Hombach A., Kruis W. (2000). Chronic inflammatory bowel disease and cancer. Hepatogastroenterology.

[52] Bermejo F., Algaba A., Guerra I., Chaparro M., De-La-Poza G., Valer P., Piqueras B., Bermejo A., García-Alonso J., Pérez M.J. (2013). Should we monitor vitamin B12 and folate levels in Crohn’s disease patients?. Scand. J. Gastroenterol..

[53] Pan Y., Liu Y., Guo H., Jabir M.S., Liu X., Cui W., Li D. (2017). Associations between Folate and Vitamin B12 Levels and Inflammatory Bowel Disease: A Meta-Analysis. Nutrients.

[54] Erzin Y., Uzun H., Celik F., Aydin S., Dirican A., Uzunismail H. (2008). Hyperhomocysteinemia in inflammatory bowel disease patients without past intestinal resections: Correlations with cobalamin, pyridoxine, folate concentrations, acute phase reactants, disease activity, and prior thromboembolic complications. J. Gastroenterol..

[55] Mowat C., Cole A., Windsor A., Ahmad T., Arnott I., Driscoll R., Mitton S., Orchard T., Rutter M., Younge L. (2011). Guidelines for the management of Inflammatory Bowel Disease in adults. Gut.

[56] Lochs H., Sobotka L. (2004). Nutritional support in inflammatory bowel disease. Basics in Clinical Nutrition.

[57] Mijač D.D., Janković G.L., Jorga J., Krstić M.N. (2010). Nutritional status in patients with active inflammatory bowel disease: prevalence of malnutrition and methods for routine nutritional assessment. Eur. J. Intern. Med..

[58] Cruz-Jentoft A.J., Baeyens J.P., Bauer J.M. (2010). European Working Group on Sarcopenia in Older People. Sarcopenia: European consensus on definition and diagnosis: Report of the European Working Group on Sarcopenia in Older People. Age Ageing.

[59] Megan P.R. (2009). Body Composition Measured by Dual-energy X-ray Absorptiometry Half-body Scans in Obese Adults. Obesity.

[60] Emerenziani S., Biancone L., Guarino M.P.L., Balestrieri P., Stasi E., Ribolsi M., Rescio M.P., Altomare A., Cocca S., Pallone F. (2017). Nutritional status and bioelectrical phase angle assessment in adult Crohn disease patients receiving anti-TNFα therapy. Dig. Liver Dis..

[61] Kalyani R.R., Corriere M., Ferrucci L., Tandon P. (2014). Age-related and disease muscle loss: the effect of diabetes, obesity, and other diseases. Lancet Diabetes Endocrinol..

[62] Barbat-Artigas S., Rolland Y., Zamboni M., Aubertin-Leheudre M. (2012). How to assess functional status: A new muscle quality index. J. Nutr. Health Aging.

[63] Goodpaster B.H., Park S.W., Harris T.B., Kritchevsky S.B., Nevitt M., Schwartz A.V. (2006). The loss of skeletal muscle strength, mass, and quality in older adults: The health, aging and body composition study. J. Gerontol. A Biol. Sci. Med. Sci..

[64] Brown A.C., Rampertab S.D., Mullin G.E. (2011). Existing dietary guidelines for Crohn’s disease and ulcerative colitis. Expert Rev. Gastroenterol. Hepatol..

[65] Hwang C., Ross V., Mahadevan U. (2014). Popular exclusionary diets for inflammatory bowel disease: The search for a dietary culprit. Inflamm. Bowel Dis..

[66] Levenstein S., Prantera C., Luzi C., D’Ubaldi A. (1985). Low residue or normal diet in Crohn’s disease: A prospective controlled study in Italian patients. Gut.

[67] Brotherton C.S., Martin C.A., Long M.D., Kappelman M.D., Sandler R.S. (2016). Avoidance of fiber is associated with greater risk of crohn’s disease flare in a 6-month period. Clin. Gastroenterol. Hepatol..

[68] Jowett S.L., Seal C.J., Pearce M.S., Phillips E., Gregory W., Barton J.R., Welfare M.R. (2004). Influence of dietary factors on the clinical course of ulcerative colitis: A prospective cohort study. Gut.

[69] Prince A.C., Myers C.E., Joyce T., Irving P., Lomer M., Whelan K. (2016). Fermentable carbohydrate restriction (low fodmap diet) in clinical practice improves functional gastrointestinal symptoms in patients with inflammatory bowel disease. Inflamm. Bowel Dis..

[70] Penagini F., Dilillo D., Borsani B., Cococcioni L., Galli E., Bedogni G., Zuin G., Zuccotti G.V. (2016). Nutrition in pediatric inflammatory bowel disease: from etiology to treatment. A systematic review. Nutrients.

[71] Società Italiana di Nutrizione Parenterale e Enterale (SINPE) (2002). Linee Guida SINPE per la Nutrizione Artificiale Ospedaliera 2002. Rivista Italiana di Nutrizione Parenterale e Enterale.

[72] Muller C., Compher D., Druyan M.E., the American Society for Parenteral and Enteral Nutrition (ASPEN) (2011). ASPEN Clinical Guidelines: Nutrition screening, assessment, and intervention in adults. J. Parenter Enteral. Nutr..

[73] Bannerjee K., Camacho-Hübner C., Babinsk K., Dryhurst K.M., Edwards R., Savage M.O. (2004). Anti-inflammatory and growth-stimulating effects precede nutritional restitution during enteral feeding in Crohn’s disease. J. Pedriatr. Gastroenterol. Nutr..

[74] Guagnozzi D., González-Castillo S., Olveira A., Lucendo A.J. (2012). Nutritional treatment in inflammatory bowel disease. An update. Rev. Esp. Enferm. Dig..

[75] Griffiths A.M., Ohlsson A., Sherman P.M., Sutherland L.R. (1995). Meta-analysis of enteral nutrition as a primary treatment of active Crohn’s disease. Gastroenterology.

[76] Zachos M., Tondeur M., Griffiths A.M. (2007). Enteral nutritional therapy for induction of remission in Crohn’s disease. Cochrane Database Syst. Rev..

[77] Dziechciarz P., Horvath A., Shamir R., Szajewska H. (2007). Meta-analysis: Enteral nutrition in active Crohn’s disease in children. Aliment. Pharmacol. Ther..

[78] Heuschkel R.B., Menache C.C., Megerian J.T., Baird A.E. (2000). Enteral nutrition and corticosteroids in the treatment of acute Crohn’s disease in children. J. Pediatr. Gastroenterol. Nutr..

[79] Forbes A., Escher J., Hébuterne X., Kłęk S., Krznaric Z., Schneider S., Shamir R., Stardelova K., Wierdsma N., Wiskin A.E. (2017). ESPEN guideline: Clinical nutrition in inflammatory bowel disease. Clin. Nutr..

[80] Nguyen G.C., Laveist T.A., Brant S.R. (2007). The utilization of parenteral nutrition during the in-patient management of inflammatory bowel disease in the United States: A national survey. Aliment. Pharmacol. Ther..

